# Supplementary material for: Atomic Structure of Type VI Contractile Sheath from Pseudomonas aeruginosa
Source: Structure. 2018 Feb 6;26(2):329–336.e3. doi: 10.1016/j.str.2017.12.005 (PMC5807055; doi:10.1016/j.str.2017.12.005)
Supplement: Document S1. Figures S1–S5 and Table S1 [file mmc1.pdf]

Structure, Volume *26*

Supplemental Information

Atomic Structure of Type VI Contractile Sheath from *Pseudomonas aeruginosa*

Osman Salih, Shaoda He, Sara Planamente, Lasse Stach, James T. MacDonald, Eleni Manoli, Sjors H.W. Scheres, Alain Filloux, and Paul S. Freemont

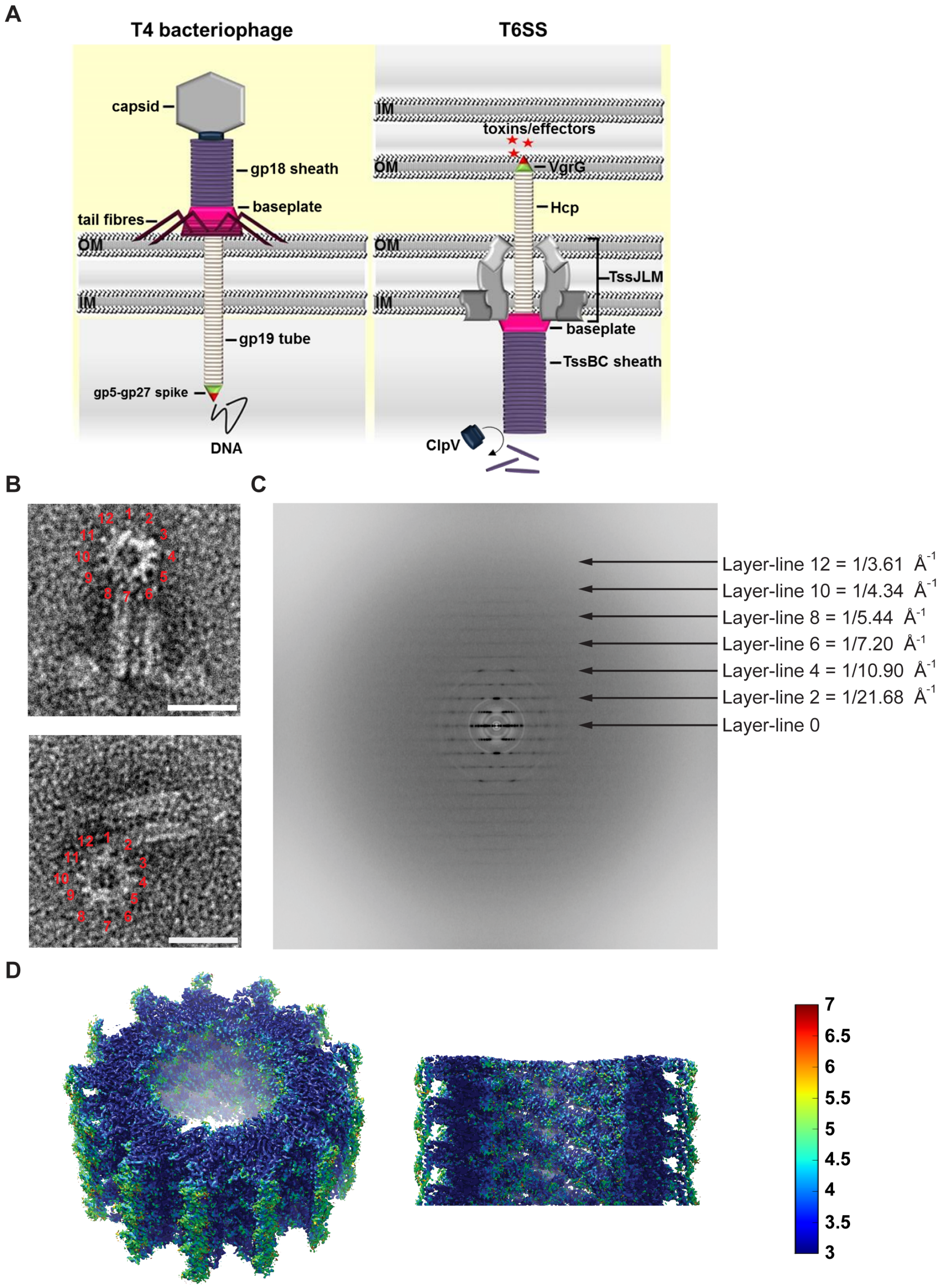

Figure S1. T6SS working model and assessing the quality of the electron microscopy data. Related to Figure 1. **A.** Working model of the T6SS assembly and its comparison with the T4 bacteriophage. The tail-like structure of the T6SS is highlighted: the Hcp tube in yellow, the VgrG spike in green and the TssBC sheath in purple. The AAA+ ATPase ClpV is shown in blue. Homologous structures found in the T4 phage are also shown and the same color code is applied. **B.** Top-views of TssB1C1 sheaths produced in *P. aeruginosa* showing 12-fold symmetry. The scale bars are 290 Å. **C.** Sum of Fourier transforms calculated from a subset of ~ 29,000 segmented sheaths in ~ 100 images showing layer lines in reciprocal space, extending up to 3.6 Å. **D.** Tilt-view and cut-away of a 162 Å long segment of the TssB1C1 sheath reconstruction. The structure is colored according to local resolution, ranging from 3 (blue) to 7 (red) Å.

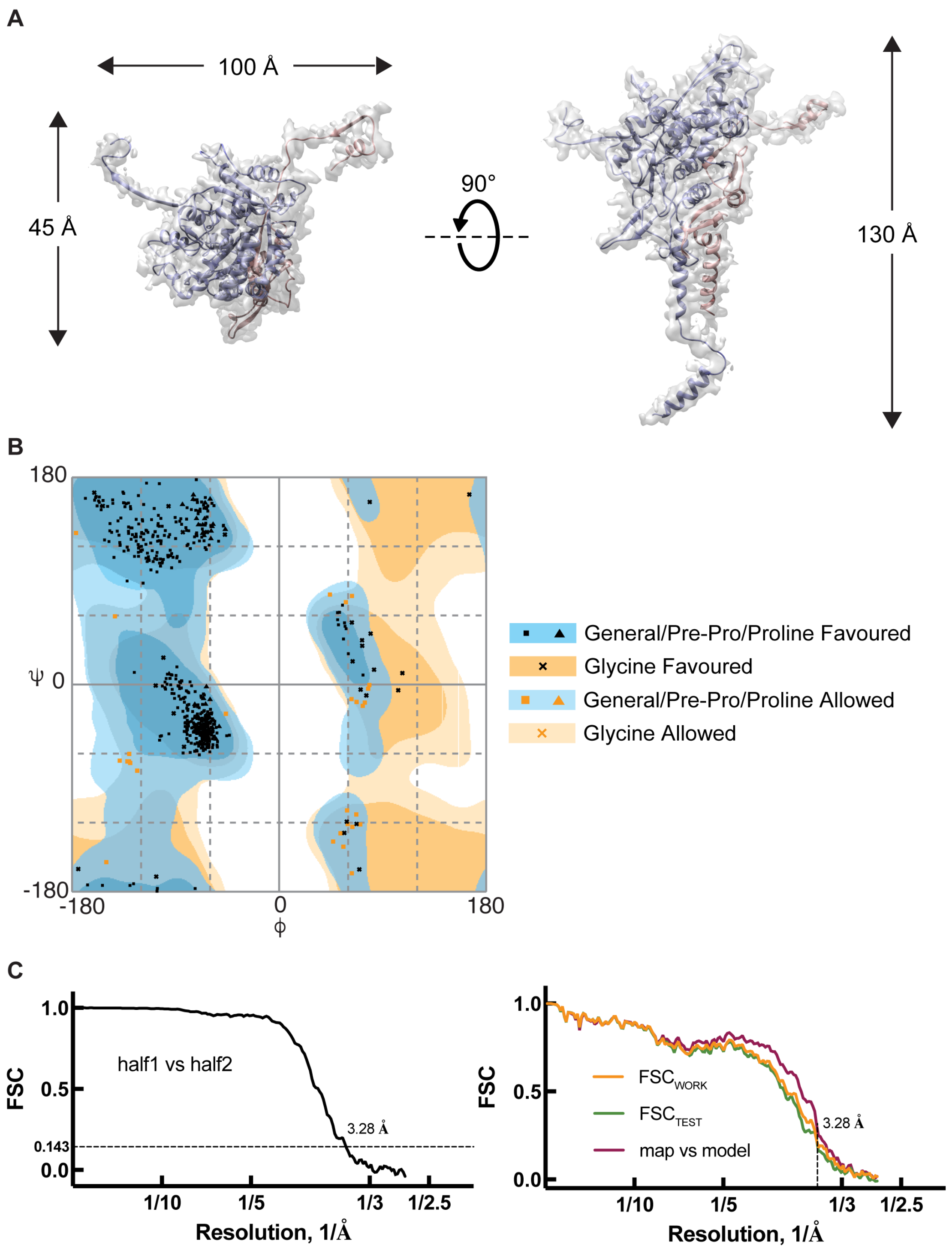

Figure S2. Atomic model of the TssB1C1 sheath. Related to Figures 1 and 2, and Table 1. **A**. Ribbon representation of the TssB1C1 heterodimer in its electron density map. Dimensions are indicated. **B**. Ramachandran plot of the TssB1C1 heterodimer. The Ramachandran plot was based on the atomic coordinates of the TssB1C1 heterodimer and was generated using the program RAMPAGE (Lovell et al., 2003). **C**. (Left) Fourier Shell Correlation (FSC) curve between the two halves of the final 3D electron density map. The resolution is 3.3 Å based on the gold-standard 0.143 FSC criterion. (Right) Cross-validation FSC_{WORK} and FSC_{TEST} curves demonstrate that an optimal weight during refinement was used to avoid over fitting. The FSC curve between the final 3D electron density map and the final refined atomic structure is also provided.

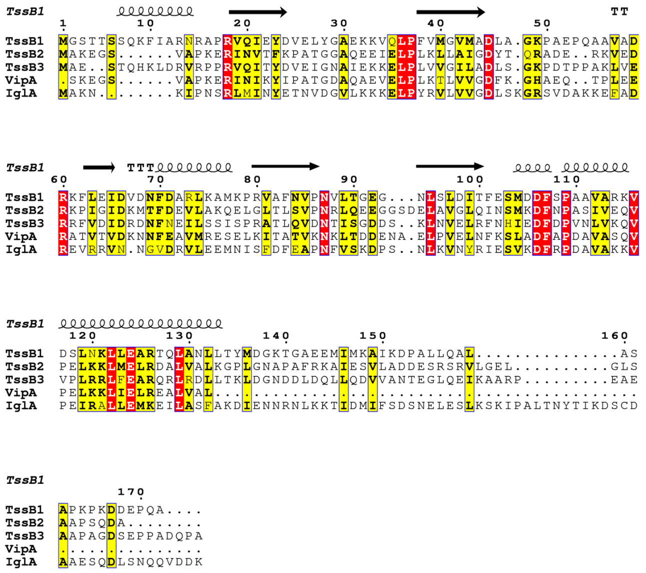

Figure S3. Sequence alignment of TssB proteins. Related to Figure 3. TssB protein sequences from *P. aeruginosa* were extracted from the Pseudomonas Genome Database; VipA and IglA sequences were retrieved from NCBI (https://www.ncbi.nlm.nih.gov/). Sequence alignments have been performed using MAFFT and the corresponding image generated using the web server ESPript 3 (http://espript.ibcp.fr). Secondary structure elements based on the *P. aeruginosa* TssB1 structure are displayed above the alignment.

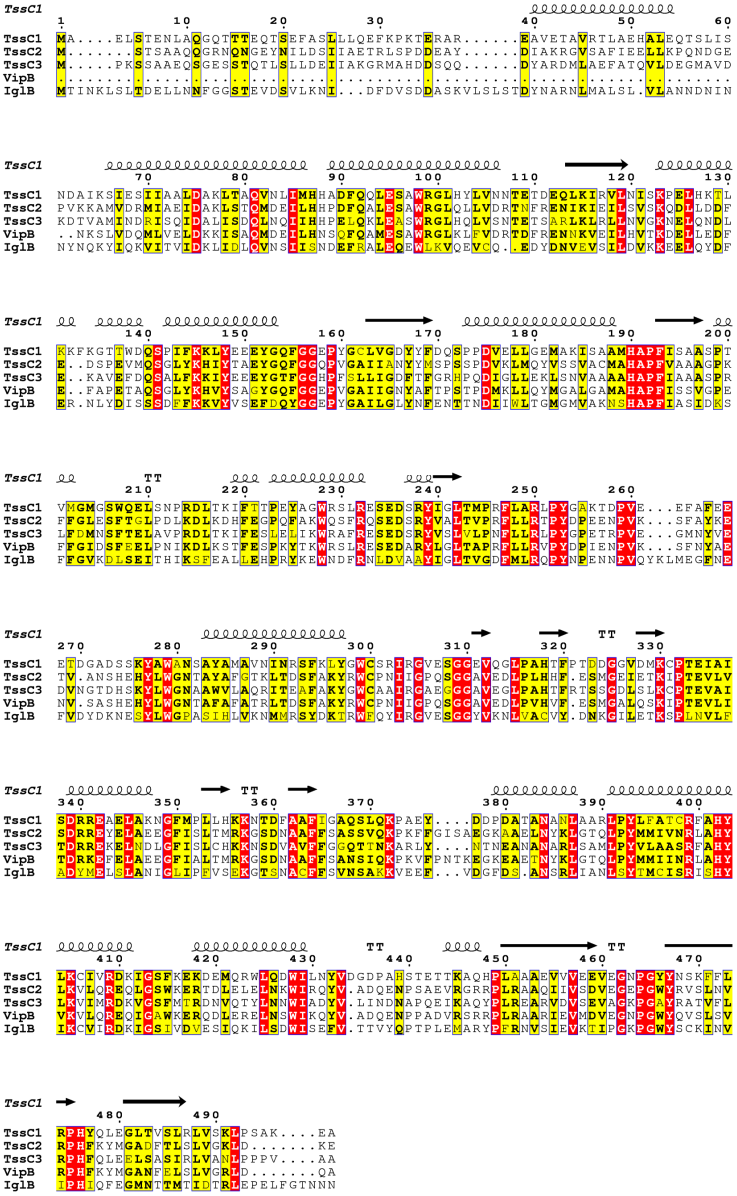

Figure S4. Sequence alignment of TssC proteins. Related to Figure 3. TssC protein sequences from *P. aeruginosa* were extracted from the Pseudomonas Genome Database; VipB and IglB sequences were retrieved from NCBI (https://www.ncbi.nlm.nih.gov/). Sequence alignments have been performed as described in Figure S3. Secondary structure elements based on the *P. aeruginosa* TssC1 structure are displayed above the alignment.

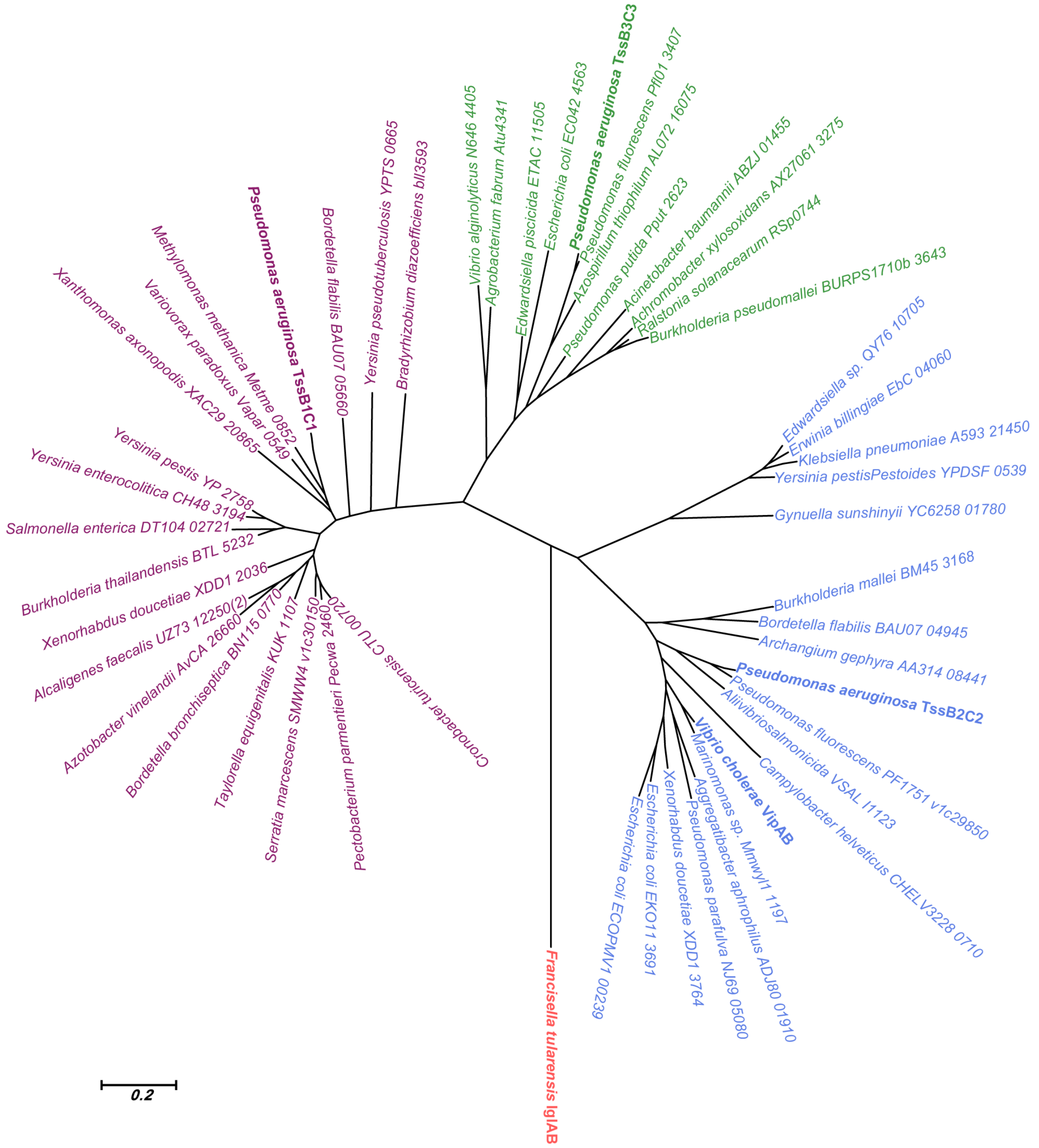

Figure S5. Phylogenetic analysis of T6SS sheath proteins. Related to Figures 3 and 4. The maximum-likelihood phylogenetic tree has been generated from 77 concatenated sequences of TssB-TssC. Concatenated sequences of the sheath proteins from *P. aeruginosa* (TssB1C1, TssB2C2 and TssB3C3), *V. cholerae* (VipAB) and *F. tulerensis* (IglAB) are highlighted in bold. The tree is drawn to scale, with branch lengths measured in the number of substitutions per site. All positions containing gaps and missing data were eliminated. Evolutionary analyses were conducted in MEGA7 (Kumar et al., 2016).

Table S1. Comparison of different sheath structures including the TssB1C1 models. Related to Figure 4.

	Sheath Interactions			Intra-strand Interactions			Inter-strand Interactions		
	Total Interface Area ($Å^2$)	Total No. H-Bonds	Total No. Salt Bridges	Total Interface Area ($Å^2$)	Total No. H-Bonds	Total No. Salt Bridges	Total Interface Area ($Å^2$)	Total No. H-Bonds	Total No. Salt Bridges
TssB1/TssC1 (13-mer)	9877.2	63	42	2671	21	15	3876.4	24	19
VipA/VipB (12-mer)	8756.7	87	40	2670.2	32	18	2593.5	20	8
IglA/IglB (12-mer)	9012.1	39	25	2895	15	8	2816.1	12	5
Contracted (12-mer)	9460.7	71	34	2565.5	21	10	3667.4	23	13
Extended (12-mer)	9732.1	69	32	3635.5	30	11	2637.5	13	8
