# Supplementary material for: Atomic Structure of Type VI Contractile Sheath from Pseudomonas aeruginosa
Source: Structure. 2018 Feb 6;26(2):329–336.e3. doi: 10.1016/j.str.2017.12.005 (PMC5807055; doi:10.1016/j.str.2017.12.005)
Supplement: Document S2. Article plus Supplemental Information [file mmc3.pdf]

Short Article

Structure

Atomic Structure of Type VI Contractile Sheath from *Pseudomonas aeruginosa*

Graphical Abstract

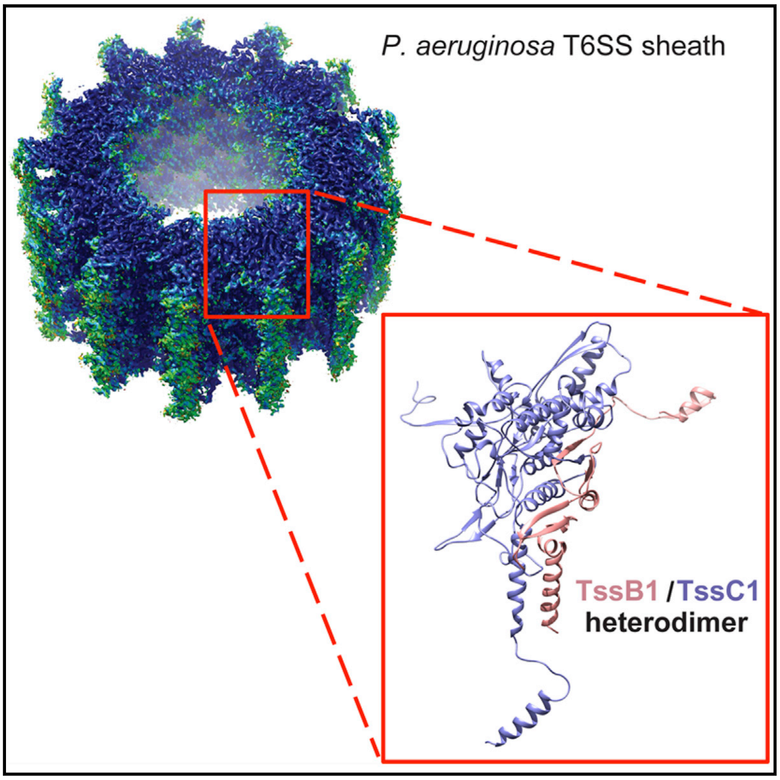

Highlights

- We solved a T6SS sheath structure from *Pseudomonas aeruginosa* (group 3 T6SS^{i})
- Comparisons between T6SS groups suggest a conserved sheath contraction mechanism
- Extended-state model led to proposal of a spring-like sheath contraction mechanism

Authors

Osman Salih, Shaoda He, Sara Planamente, ..., Sjors H.W. Scheres, Alain Filloux, Paul S. Freemont

Correspondence

a.filloux@imperial.ac.uk (A.F.), p.freemont@imperial.ac.uk (P.S.F.)

In Brief

Salih et al. present the atomic structure of a contracted sheath of a type VI secretion system, revealing unique features. Comparison with other systems suggests a conserved sheath contraction mechanism among T6SS groups. Modeling the extended state, the authors suggest a coiled-spring-like mechanism for T6SS sheath contraction.

Salih et al., 2018, Structure *26*, 329–336
February 6, 2018 © 2017 The Author(s). Published by Elsevier Ltd.
https://doi.org/10.1016/j.str.2017.12.005

Structure
Short Article

Atomic Structure of Type VI Contractile Sheath from *Pseudomonas aeruginosa*

Osman Salih,[1,4] Shaoda He,[2,4] Sara Planamente,[3] Lasse Stach,[1] James T. MacDonald,[1] Eleni Manoli,[3] Sjors H.W. Scheres,[2] Alain Filloux,[3,*] and Paul S. Freemont[1,5,*]
[1]Section of Structural Biology, Department of Medicine, Imperial College London, London SW7 2AZ, UK
[2]MRC Laboratory of Molecular Biology, Cambridge CB2 0QH, UK
[3]MRC Centre for Molecular Bacteriology and Infection (CMBI), Department of Life Sciences, Imperial College London, London SW7 2AZ, UK
[4]These authors contributed equally
[5]Lead Contact
*Correspondence: a.filloux@imperial.ac.uk (A.F.), p.freemont@imperial.ac.uk (P.S.F.)
https://doi.org/10.1016/j.str.2017.12.005

SUMMARY

Pseudomonas aeruginosa **has three type VI secretion systems (T6SSs), H1-, H2-, and H3-T6SS, each belonging to a distinct group. The two T6SS components, TssB/VipA and TssC/VipB, assemble to form tubules that conserve structural/functional homology with tail sheaths of contractile bacteriophages and pyocins. Here, we used cryoelectron microscopy to solve the structure of the H1-T6SS** ***P. aeruginosa*** **TssB1C1 sheath at 3.3 Å resolution. Our structure allowed us to resolve some features of the T6SS sheath that were not resolved in the** ***Vibrio cholerae*** **VipAB and** ***Francisella tularensis*** **IglAB structures. Comparison with sheath structures from other contractile machines, including T4 phage and R-type pyocins, provides a better understanding of how these systems have conserved similar functions/mechanisms despite evolution. We used the** ***P. aeruginosa*** **R2 pyocin as a structural template to build an atomic model of the TssB1C1 sheath in its extended conformation, allowing us to propose a coiled-spring-like mechanism for T6SS sheath contraction.**

INTRODUCTION

The type VI secretion system (T6SS) is a contractile injection machine widely distributed among Gram-negative bacteria. Bacteria use the T6SS to directly deliver effectors into bacteria or eukaryotic cells (Cianfanelli et al., 2016). T6SSs are therefore crucial for virulence of many bacterial pathogens, including *Pseudomonas aeruginosa*, which has three gene clusters (H1-, H2-, and H3-T6SSs) encoding them (Sana et al., 2016).

The T6SS apparatus consists of around 13 core components and resembles bacteriophage tails (Ho et al., 2014). The current model of the T6SS suggests that its core components form three subassemblies: (1) a membrane anchoring complex (Durand et al., 2015), (2) a baseplate (Brunet et al., 2015; Planamente et al., 2016), and (3) a tail-like structure. The last is the most distinctive feature of the T6SS and is made of Hcp, VgrG, and a sheath-like structure. The T6SS sheath comprises two proteins, TssB (VipA/IglA) and TssC (VipB/IglB), which form tubular structures similar to the T4 phage gp18 sheath (Leiman et al., 2009; Lossi et al., 2013; Clemens et al., 2015; Kudryashev et al., 2015).

The T4 phage sheath assembles in a high-energy extended state from the baseplate around the rigid gp19 tube serving as a scaffold. Upon receiving cell-contact-dependent stimuli, the basal complex undergoes conformational changes that trigger sheath contraction, injecting DNA into target cells (Yap and Rossmann, 2014). It is thought that mechanisms of sheath assembly and contraction are conserved among contractile injection systems, including T6SSs, R-type pyocins, and phage-like protein translocation structures (Kube and Wendler, 2015). By comparison with phage assembly, one can propose that in the T6SS the VgrG spike is surrounded by baseplate-like wedges upon which Hcp hexamers assemble to form a rigid tail tube, while the TssBC sheath wraps around the tube in an extended conformation. Upon sheath contraction the T6SS fires Hcp/VgrG and bound toxins and is subsequently recycled by the AAA+ ATPase ClpV, which binds the contracted sheath to disassemble it (Figure S1A; Kapitein et al., 2013; Kube et al., 2014). The latter feature is not conserved in phages since no tail recycling is needed. Thus, T6SS activity relies on the proper assembly and contraction of its TssBC sheath, and atomic details of such structures are crucial to unravel the mechanism of action.

T6SS sheath dynamics have been studied *in vivo* using live imaging (Basler and Mekalanos, 2012; Basler et al., 2012; Vettiger et al., 2017), and cryoelectron microscopy (cryo-EM) structures of T6SS sheaths from *Vibrio cholerae* and *Francisella tularensis* are known (Clemens et al., 2015; Kudryashev et al., 2015). However, mechanisms associated with T6SS sheath contraction remain speculative and a robust model of an extended conformation of the T6SS sheath has not been reported.

Three distinct T6SS subtypes exist (Russell et al., 2014), T6SS^{i}, in which most proteobacterial T6SSs are found, including *V. cholerae* and *P. aeruginosa*; T6SS^{ii} for the *Francisella* T6SS; and T6SS^{iii} for Bacteroidetes systems. Several groups are phylogenetically identified within the T6SS^{i} subtype; the *P. aeruginosa* H2-T6SS falls into group 1 with the *V. cholerae* T6SS, while H3-T6SS is in group 4, and H1-T6SS is in group 3. Here, we report the atomic structure of the TssB1C1 sheath encoded by the

This is an open access article under the CC BY license (http://creativecommons.org/licenses/by/4.0/).

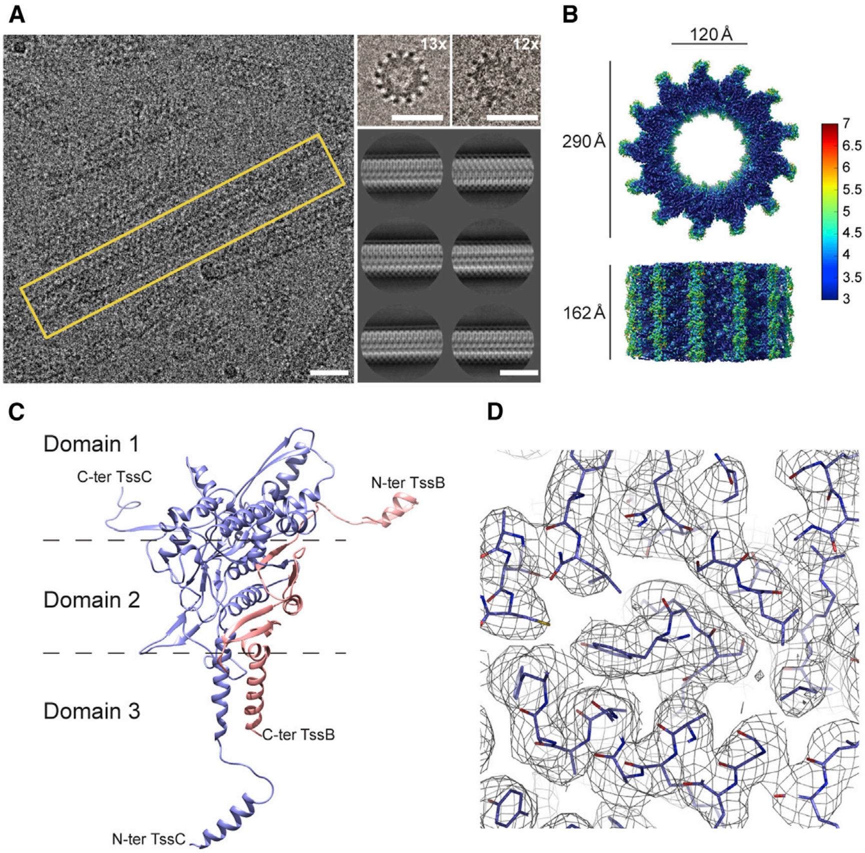

Figure 1. Cryo-EM of *P. aeruginosa* TssB1C1 Sheath

(A) EM field of TssB1C1 sheaths embedded in vitreous ice showing tubular assemblies with a single sheath tube highlighted in a yellow box (left). Top views of sheaths showing a cogwheel-like structure with a mix of 12- and 13-fold symmetries (top right). Two-dimensional class averages of TssB1C1 sheaths are presented (bottom right) and appear asymmetric across their meridians, indicating that the dataset is dominated by 13-fold symmetric helical segments. All images are shown with no symmetry imposed. The scale bars represent 290 Å.

(B) Cryo-EM density map of a 162 Å long segment of the TssB1C1 sheath determined at 3.3 Å resolution. Top and side views are shown and the structure is colored according to local resolution, ranging from 3 (blue) to 7 (red) Å.

(C) Ribbon representation of the atomic model of the TssB1C1 heterodimer structure wherein TssB1 is in pink and TssC1 is in purple. Termini are labeled and dashed lines roughly delineate domains 1, 2, and 3.

(D) Close-up of the atomic structure built into the electron density map contoured at 2σ, illustrating the level of detail visible in the cryo-EM reconstruction.

See also Figures S1 and S2.

H1-T6SS cluster from *P. aeruginosa*. Our 3.3 Å cryo-EM structure reveals unprecedented details of a T6SS^{i}/group 3 sheath, allowing comparison with structures from *V. cholerae* (T6SS^{i}/group 1) and *F. tularensis* (T6SS^{ii}), as well as with other contractile injection machines. Finally, we built an atomic model of the TssB1C1 sheath in its extended conformation. Our structures enable comprehensive comparisons between homologous systems, providing insights into sheath assembly/disassembly and the conformational transitions during sheath contraction.

RESULTS AND DISCUSSION

Cryo-EM Structure of the TssB1C1 Sheath of *Pseudomonas aeruginosa*

P. aeruginosa TssB1 and TssC1 proteins produced in *Escherichia coli* spontaneously assemble (1:1 stoichiometry) to form tubes (Lossi et al., 2013). Here, we optimized the purification conditions of the recombinant TssB1C1 complex by using a more gentle cell lysis method and screening pH and salt concentration to obtain long, unbroken tubes to pursue cryo-EM analysis. TssB1C1 tubes vary in length (300–5,000 Å) and have an outer diameter of 290 Å and an inner channel width of 120 Å. Side views of the sheath reveal a diagonally striated pattern that is characteristic of helical assemblies. Top views of short tubes are cogwheel-like in appearance and predominantly show 13-fold symmetry, although we also observed a few 12-fold symmetric tubes (Figure 1A). Since 12- and 13-fold symmetries are present, and given that all known contractile systems have 12-fold symmetry, we assume the 13-fold symmetry of the TssB1C1 sheath is due to sample preparation. This is further supported by negative-stain EM data showing that TssB1C1 sheaths produced in the native host *P. aeruginosa* have 12-fold symmetry (Figure S1B).

To determine an atomic model of TssB1C1, we froze tubes purified from *E. coli* and collected a cryo-EM dataset. The 3D structure of TssB1C1 was obtained at 3.3 Å resolution using helical reconstruction within RELION (He and Scheres, 2017; Figures 1B, S1, and S2). The atomic model of TssB1C1 was built using a combination of *de novo* and molecular replacement methods. For the latter, we used the VipAB structure (PDB: 3J9G; Kudryashev et al., 2015) as a search model, and once docked into the TssB1C1 sheath cryo-EM density map, non-conserved residues were changed. *De novo* techniques were used to manually rebuild a number of loops and turns to give our initial TssB1C1 heterodimer model. Compared with the VipAB structure, additional residues could be built toward the termini of the two protein chains. Initially, the repeating unit of the TssB1C1 heterodimer alone was refined against the electron density map. Then, the larger symmetrical assembly was refined against the EM map. The final 3D model of the 13-fold symmetric TssB1C1 sheath has a helical rise and twist of 20.2 Å and 27.7°, respectively, and presents a right-handed six-start helical assembly composed of discrete densities corresponding to the heterodimers. The helical organization of TssB1C1 protomers results in 13 parallel stiles that run along the sheath length and correspond to the spokes of the cogwheels in projection (Figure 1A). Side and cut-away views (Figures 1B and S1) of the reconstruction show the 13-mer sheath as being composed of non-planar

Table 1. Cryo-EM Data Collection, Refinement, and Validation

	13-Fold Symmetry TssB1C1 (PDB: 5N8N)
Data Collection	
Electron microscope	FEI Titan Krios
Detector	FEI Falcon II
Voltage (kV)	300
Defocus range (μm)	1.0–2.5
Electron dose (e^{-} $Å^{-2}$)	30
Number of collected particles	71,264
Pixel size (Å)	1.34
Model Composition	
Non-hydrogen atoms	70,035
Protein residues	8,895
Refinement	
Resolution (Å)	3.3
Map sharpening B factor ($Å^2$)	−75.8
Average B factor ($Å^2$)	68.8
R factor	0.3381
Average Fourier shell correlation	0.8336
RMS Deviations	
Bonds (Å)	0.019
Angles (°)	1.336
Ramachandran Plot	
Favored (%)	95.59
Outliers (%)	0
Validation	
MolProbity score	2.06 (100th percentile)
All-atom clash score	17.99 (97th percentile)
Favored rotamers (%)	95.34
Poor rotamers (%)	0.81

See also Figure S2.

discs, each comprising 6.5 heterodimers for every 360° turn of the sheath due to a 3.36 Å axial rise difference between units.

All solved sheath structures are 12-fold helical tubes with outer diameters of 240–330 Å and channel widths of 100–120 Å in their contracted states. For a reliable comparison with related sheath structures, we generated a 12-fold symmetry model of *P. aeruginosa* TssB1C1. We used the homologous *V. cholerae* VipAB structure (PDB 3J9G) as a template and potential packing differences with the obtained 13-fold structure were investigated. As anticipated, the 12-fold symmetric TssB1C1 sheath model is very similar to the *V. cholerae* VipAB assembly with a helical rise and twist of 22.1 Å and 29.6°, respectively. The 12-mer model of TssB1C1 has a slightly larger interface area of 9,461 $Å^2$ compared with the VipAB assembly (8,757 $Å^2$). The main difference between the 12- and the 13-mer TssB1C1 assemblies is the buried surface area (13-mer 9,877 $Å^2$ and 12-mer 9,461 $Å^2$; Table S1), which is due to the difference in subunit packing in these assemblies.

Although a T6SS tail-tube/sheath complex has not been isolated yet, it is thought that the inner channel of the sheath can accommodate a rigid tube made of stacked Hcp hexamers. The symmetry and dimensions of the inner tube protein Hcp are similar to those of known contractile systems, namely gp19 in T4 phage and the pyocin tube protein (Kostyuchenko et al., 2005; Ge et al., 2015). Moreover, in R-type pyocins, the inner tube binds to the sheath mainly through electrostatic interactions, which has also been shown for the Hcp-sheath interaction in the T6SS of *Edwardsiella tarda* (Jobichen et al., 2010).

Altogether these findings strengthen the idea that despite differences in amino acid composition and sheath dimensions, tails of contractile machines have a conserved helical assembly and related mechanisms.

Atomic Details of the TssB1C1 Assembly

The final model of the TssB1C1 heterodimer (Figure 1C) obtained from the EM map has good stereochemistry and refinement statistics (Figures 1D and S2; Table 1). We were unable to trace a small number of residues located at the outer extremity of the sheath and termini (residues M1–S3 and M136–A172 for TssB1 and M1–A37 for TssC1), as the EM density is less resolved. However, we were able to trace an additional TssC1 N-terminal α helix (H1; residues E39–E54) and loop (residues Q55–K65) in the outer sheath layer (Figure 2) that were not resolved in previous sheath structures (Figure 3; Clemens et al., 2015; Kudryashev et al., 2015).

The TssB1C1 heterodimer within the helical assembly of the sheath forms an intricate packing arrangement that is organized in three domains (domains 1, 2, and 3; Figure 1C). Two β strands of TssC1 (β11 and β12) are joined by two donor β strands, one from a neighboring TssC1 subunit (β13) within the same protofilament and the other from an adjacent TssB1 subunit (β1) in a different protofilament, but within the same disc, to form a β sheet interaction (handshake interaction) at the inner layer of the sheath (domain 1; Figure 2B*i*). The protruding arms of the heterodimer contribute in a similar manner to the folds of nearby subunits, i.e., TssB1 N-terminal β strand and TssC1 C-terminal β strand form inter- and intra-protofilament interactions, respectively. Two β strands of TssB1 (β2 and β3) interlaced with five β strands from TssC1 (β1–β4 and β8) form a twisted seven-stranded β sheet flanked by α helices in the middle layer of the sheath wall (domain 2; Figure 2B*ii*). Two α helices (residues D117–L133 of TssB1 and S66–M86 of TssC1) and a third α helix (residues E39–E54 of TssC1) form a tri-helical bundle (domain 3; Figure 2B*iii*). This third α helix is in an extended conformation in our TssB1C1 sheath, radiating away from its originating heterodimer and interacting with an adjacent heterodimer directly below that belongs to a different protofilament, but is positioned within the same stile of the helical assembly (Figure 2).

Interface interactions between the TssB1 and the TssC1 proteins observed here are very similar to those demonstrated between homologous TssB and TssC subunits, where the N terminus of TssC and a C-terminal α helix of TssB are important for the TssB-TssC interaction (Zhang et al., 2013; Clemens et al., 2015; Kudryashev et al., 2015). There are very few changes in interactions within the TssB1C1 heterodimer in the 13-mer structure versus the 12-mer model (Table S1). All major inter-protomer contacts are maintained. This may explain why TssB1C1 can

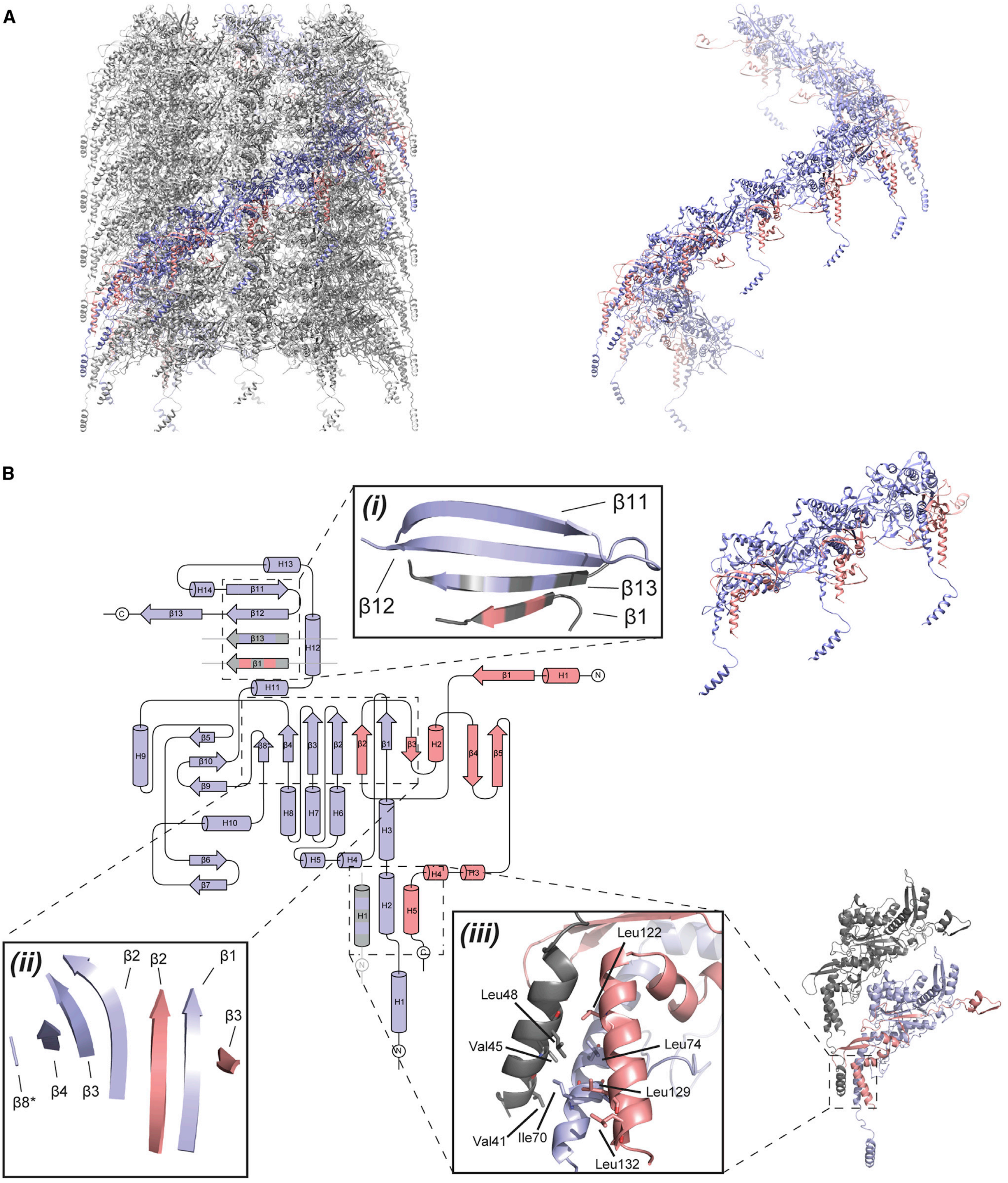

Figure 2. Inter-protomer Interactions of the TssB1C1 Heterodimer within the Sheath

(A) TssB1C1 heterodimers from the same protofilament are colored (TssB1 in pink and TssC1 in purple) in the context of the whole sheath (gray; left) and highlighted to show one 360° helical turn (right).

(B) Schematic representation of a TssB1C1 heterodimer color coded as described above. (*i*) Handshake interaction responsible for tube formation. (*ii*) β sheet essential for maintaining the structural integrity of the TssB1C1 heterodimer. (*iii*) Tri-helical vertical packing interaction along stiles stabilized by hydrophobic interactions. See also Figure S2.

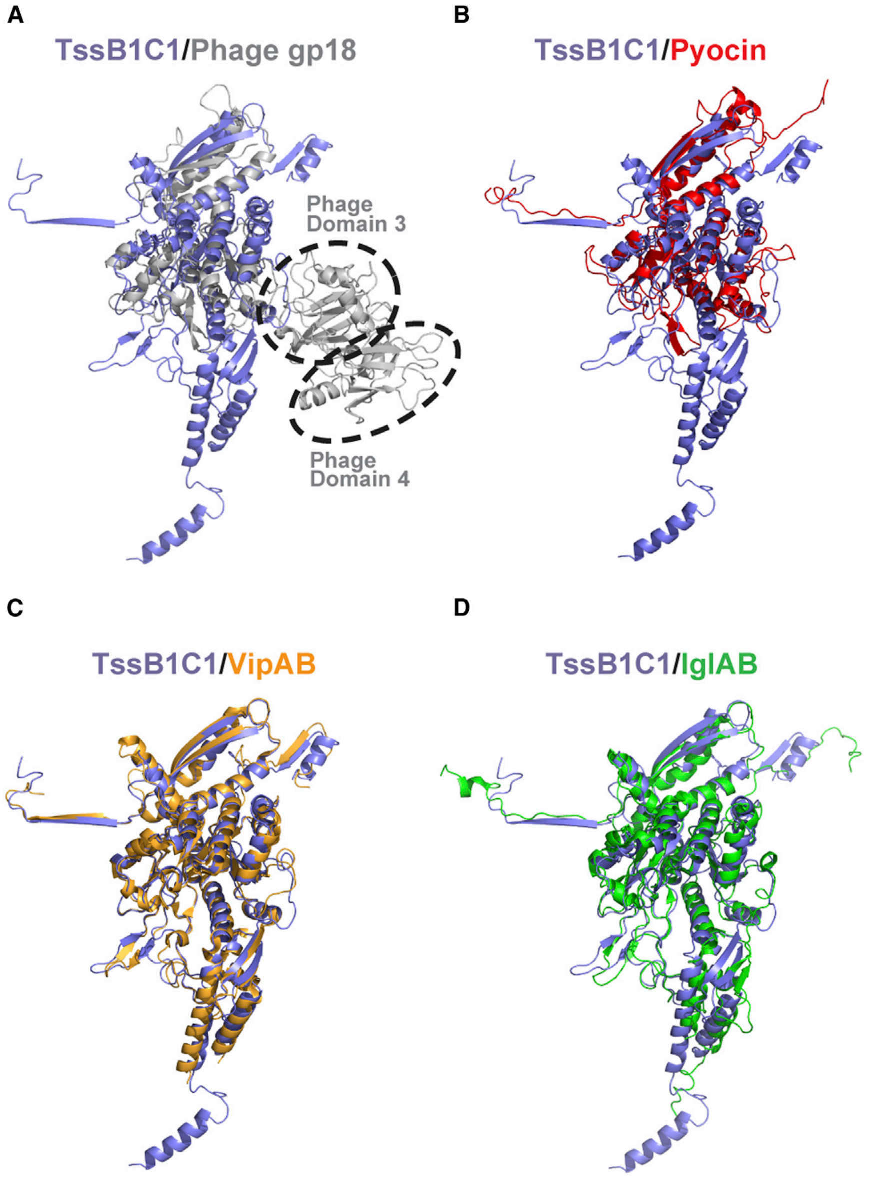

Figure 3. Structural Comparison between TssB1C1 and Other Contractile Systems

Superposition of the ribbon structure of TssB1C1 (purple) with gp18 (gray; root-mean-square deviation [RMSD] 3.40 Å for 214 Cα) and with the pyocin sheath subunit (red; RMSD 2.59 Å for 268 Cα) is shown in (A and B), respectively. Domains that are unique to the phage protein are circled. Superposition of TssB1C1 with VipAB (orange; RMSD 1.34 Å for 535 Cα) and with IglAB (green; RMSD 1.93 Å for 482 Cα) is shown in (C and D), respectively. See also Figures S3–S5.

assemble in both conformations *in vitro*. The tri-helical bundle interactions undergone by the TssC1 α helix H1 resolved in the TssB1C1 structure are also maintained in the 12-mer model. Moreover, this helix is conserved in *V. cholerae* VipB, *Francisella novicida* IglB, and *P. aeruginosa* TssC2 (H2-T6SS) and TssC3 (H3-T6SS) sheath proteins (Figures S3 and S4). This suggests that the tri-helical vertical packing interactions along stiles we observe for *P. aeruginosa* TssB1C1 tubes is common to a number of contractile systems.

Sheaths of Contractile Systems Share a Common Assembly

The T6SS sheath subunit is thought to be evolutionarily related to the T4 phage sheath protein gp18 (PDB 3FOA, 3FOH, 3FOI, and 3J2N; Aksyuk et al., 2009; Fokine et al., 2013). The fold of the TssB1C1 heterodimer resembles gp18 with various insertions and deletions. Although protein sequence-based alignments only showed homology between TssC and gp18 (Lossi et al., 2013), the structural overlay between gp18 and the TssB1C1 heterodimer shows that domains 1 and 2 are conserved (Figure 3A).

Major differences between these two sheath subunits are domains 3 and 4. In the T4 phage, domains 3 and 4 are located at the outer sheath layer. In the T6SS sheath, the insertion of domain 3 into domain 2 and the related angle are different compared with T4 phage (Figure 3A) and thus have an impact on the outer sheath layer. Studies from *V. cholerae* showed that the ATPase ClpV targets the N-terminal helix of VipB (TssC) located at the outer layer of the contracted sheath (domain 3; Pietrosiuk et al., 2011; Kube et al., 2014). Therefore, the divergence of the outer layers of T6SS sheaths, compared with those of contractile phage tails, may be related to their ClpV-specific recycling ability. Supporting this is the absence of domain 3 in the *P. aeruginosa* R2 pyocin sheath (PDB: 3J9Q and 3J9R), which consists of only two domains (domains 1 and 2; Figure 3B). Similar to contractile phages, R-type pyocins are also not recycled.

Comparison of TssBC sheath sequences from the three *P. aeruginosa* T6SSs (H1-, H2-, and H3-T6SS) shows a high degree of sequence similarity, with sequence identity of 33% between TssB1C1, TssB2C2, and TssB3C3. The main differences are within loop regions, suggesting that the assembly of *P. aeruginosa* sheaths from different clusters is highly conserved. Additional N- and C-terminal deletions of TssB2 and TssC2, respectively, compared with TssB1C1 (Figures S3 and S4) may affect TssB2C2 sheath assembly/stability since these deletions occur near tube-forming handshake interactions. This raises the question about possible differences in assembly/contraction mechanism between these systems within *P. aeruginosa*. One possibility is that the T6SS sheaths have subtle differences in their assembly/contraction efficiency, which may represent redundancy within

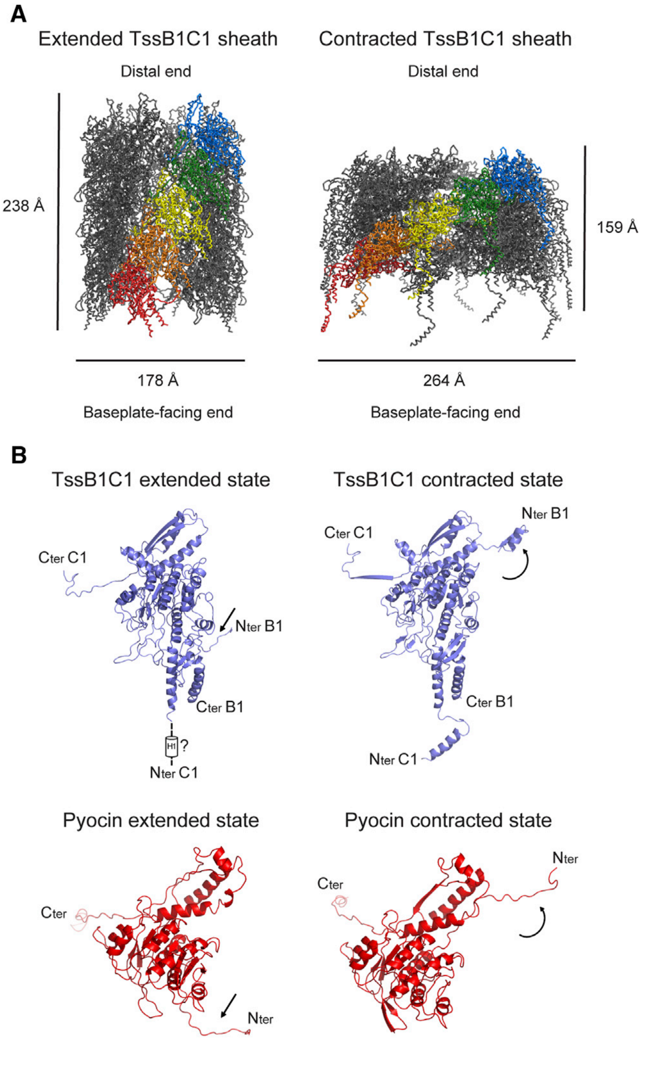

Figure 4. Computational Models of the T6SS TssB1C1 Sheath with 12-Fold Symmetry in Both Extended and Contracted Conformations

(A) Ribbon representations of the TssB1C1 sheath in an extended (left) and a contracted (right) conformation. The models are displayed as ribbons with one protofilament each in rainbow colors. The orientation of the sheaths and their dimensions are indicated. Both sheath sizes are arbitrary, each consisting of 30 whole TssB1C1 subunits.

(B) Ribbon representations of the TssB1C1 heterodimer (purple) in the extended (top left) and contracted (top right) conformations of the sheath and the R2 pyocin unit (red; PDB: 3J9Q and 3J9R) in the extended (bottom left) and contracted (bottom right) states of the pyocin sheath. The TssC1 N terminus in the extended model is indicated with dashed lines. Regions undergoing conformational changes from extended to contracted states are indicated with arrows.

See also Table S1 and Movie S1.

P. aeruginosa to maintain killing effectiveness under different conditions or targets.

T6SS Sheath Contraction: A Coiled-Spring-like Mechanism

Upon a trigger, the sheath rapidly contracts into a low-energy state injecting the Hcp tube/VgrG spike and associated toxins into target cells. Mechanisms associated with sheath contraction have been described for T4 phage tails and R-type pyocins whose extended and contracted conformers have been determined by cryo-EM (Leiman et al., 2004; Kostyuchenko et al., 2005; Ge et al., 2015).

Isolating T6SS sheaths in their extended state has not been successful to date. We therefore generated a thermodynamically stable model of an extended TssB1C1 tube with 12-fold symmetry to study the contraction event of the T6SS sheath. Molecular modeling was based on the extended structure of *P. aeruginosa* R2 pyocin, representing the closest homolog to TssB1C1, compared with gp18 (Figure 3B).

The extended TssB1C1 model is a helical assembly with a rise and twist of 38.2 Å and 18.5°, respectively (Figure 4A). This is in agreement with the molecular architecture and helical parameters observed in the extended conformation of the T6SS sheath obtained from the 3D volume of the subtomogram average from *Myxococcus xanthus* cells (Chang et al., 2017). During contraction, a 45° rotation clockwise about an axis perpendicular to the helical axis occurs, as well as translational shifts, which altogether result in 33% shortening and 48% widening of the sheath, with concomitant 42% decrease in helical rise and 60% increase in helical twist. Also, a slight increase in channel width is observed from 85 to 110 Å for extended and contracted models, respectively. Remarkably, the *M. xanthus* TssBC sheath shortens by the same amount as our TssB1C1 assembly during contraction (Chang et al., 2017), similar to that undergone by VipAB tubes (Basler et al., 2012). Upon TssB1C1 sheath contraction, the total buried surface area contributed by intra-strand interactions between heterodimers decreases, whereas inter-strand interactions increase (Table S1).

Morphing between both conformations of the T6SS sheath demonstrates that the transition from extended to contracted state is similar to that of a coiled-spring motion involving the movement of sheath subunits as rigid bodies (Movie S1). Our TssB1C1 modeling shows a mechanism similar to that described for R2 pyocins despite the difference in domain conservation (Ge et al., 2015). The protruding arms of TssB1C1 that form part of the handshake interactions with neighboring subunits act as molecular hinges facilitating this transition (Figure 4).

While extensive subunit movements are observed upon contraction, sheath subunits undergo only negligible changes. Clashes were observed between neighboring TssB1C1 subunits due to the extra TssC1 N-terminal α helix and loop (residues R38–I64; domain 3), which are not present in the R2 pyocin. Therefore, this region remains difficult to model accurately without prior positional knowledge. In our previous study, we showed that the *P. aeruginosa* TssC1-ClpV1 interaction differs with what has been described for *V. cholerae* (Forster et al., 2014). In the latter, the N terminus of VipB (TssC) is thought to be inaccessible for ClpV in the extended conformation of the sheath, preventing thus premature sheath disassembly (Pietrosiuk et al., 2011; Kapitein et al., 2013; Kube et al., 2014). Therefore, these differences highlighted between the *P. aeruginosa* H1-T6SS sheath-ClpV versus the *V. cholerae* sheath-ClpV binding mode do not allow any positioning of the N terminus of TssC1 in the extended model.

From our modeling, we estimate a −9.7 kcal/mol difference in free energy between the extended and the more stable contracted sheath, which is comparable with that calculated for R2 pyocins

at −12 kcal/mol (Ge et al., 2015). The estimate of the energy release during contraction of the T4 phage sheath is −25 kcal/mol (Arisaka et al., 1981) and is higher than that estimated for the TssB1C1 sheath. The contracted T4 phage sheath has more compact packing than TssB1C1 due to the size and rearrangement of the gp18 protomer, which could explain the difference in energy release. However, taking into account the size of T6SS sheaths *in situ*, which are much longer than phage tails, one can speculate that this difference in length can compensate for the global amount of energy released by the T6SS during contraction.

To estimate the total energy released during T6SS sheath contraction, the difference in free energy between the extended and the contracted states is combined with the number of subunits within a defined sheath length. The longest TssB1C1 assembly observed in our micrographs measured ∼5,000 Å in length, which, extrapolating from our modeling, would be composed of ∼1,150 sheath subunits. Therefore, the total energy released during contraction of the TssB1C1 sheath could be ∼11,000 kcal/mol, which is slightly larger than half the general estimate provided for a T6SS sheath twice as long at ∼18,000 kcal/mol (Basler, 2015).

In summary, our modeling shows that upon contraction, TssB1C1 can undergo conformational transitions similar to that described for R-type pyocins and contractile phages despite these systems being evolutionarily distant. Here we have solved the structure of a T6SS sheath from *P. aeruginosa*, a representative of group 3 T6SS[i]. Our structure reveals key features for T6SS sheaths such as the extended TssC1 N-terminal α helix that forms an inter-protofilament tri-helical bundle at the outer layer of the contracted sheath. We show that sequence variations between different T6SS groups would not affect the overall structure, suggesting that sheath contraction is a highly conserved mechanism. Based on sequence comparisons with the pyocin sheath, we modeled an extended conformation of the TssBC sheath and propose a spring-like mechanism for sheath contraction. Despite sequence conservation between T6SS sheaths, it will be important to determine whether the observed subtle differences between sheath classes (Figure S5) influence sheath stability/dynamics. Such knowledge may help in the development of targeted novel therapeutics.

STAR★METHODS

Detailed methods are provided in the online version of this paper and include the following:

- KEY RESOURCES TABLE
- CONTACT FOR REAGENT AND RESOURCE SHARING
- METHOD DETAILS
  - Expression and Purification of TssB1C1 Sheath
  - Negative-Stain Electron Microscopy
  - Cryo-Electron Microscopy and Image Processing
  - Atomic Building and Modeling
  - Bioinformatic Analysis
- DATA AND SOFTWARE AVAILABILITY

SUPPLEMENTAL INFORMATION

Supplemental Information includes five figures, one table, and one movie and can be found with this article online at https://doi.org/10.1016/j.str.2017.12.005.

ACKNOWLEDGMENTS

We thank Shaoxia Chen for use of the Electron Microscopy Facility at the MRC-LMB, Cambridge, and Christos Savva together with Sonja Welsch for assistance with data collection. We also thank DLS for access to beamline M02 (EM13212-1), as well as Alistair Siebert and Sacha De Carlo for help with data collection. Funding was by MRC UK (MR/K001930/1 and MC_UP_A025_1013).

AUTHOR CONTRIBUTIONS

P.S.F., A.F., S.P., and O.S. conceived, designed, and interpreted experiments; E.M., S.P., O.S., and L.S. carried out biochemical experiments; O.S., S.H., and S.H.W.S. carried out EM reconstructions; O.S. and L.S. carried out atomic building and refinement; J.T.M. carried out computational modeling; S.P., P.S.F., O.S., and A.F. wrote the paper.

Received: July 4, 2017
Revised: September 28, 2017
Accepted: December 6, 2017
Published: January 4, 2018

REFERENCES

Adams, P.D., Afonine, P.V., Bunkoczi, G., Chen, V.B., Davis, I.W., Echols, N., Headd, J.J., Hung, L.W., Kapral, G.J., Grosse-Kunstleve, R.W., et al. (2010). Phenix: a comprehensive python-based system for macromolecular structure solution. Acta Crystallogr. D Biol. Crystallogr. *66*, 213–221.

Aksyuk, A.A., Leiman, P.G., Kurochkina, L.P., Shneider, M.M., Kostyuchenko, V.A., Mesyanzhinov, V.V., and Rossmann, M.G. (2009). The tail sheath structure of bacteriophage T4: a molecular machine for infecting bacteria. EMBO J. *28*, 821–829.

Arisaka, F., Engel, J., and Klump, H. (1981). Contraction and dissociation of the bacteriophage T4 tail sheath induced by heat and urea. Prog. Clin. Biol. Res. *64*, 365–379.

Basler, M. (2015). Type VI secretion system: secretion by a contractile nanomachine. Philos. Trans. R. Soc. Lond. B Biol. Sci. *370*, 20150021.

Basler, M., and Mekalanos, J.J. (2012). Type 6 secretion dynamics within and between bacterial cells. Science *337*, 815.

Basler, M., Pilhofer, M., Henderson, G.P., Jensen, G.J., and Mekalanos, J.J. (2012). Type VI secretion requires a dynamic contractile phage tail-like structure. Nature *483*, 182–186.

Brunet, Y.R., Zoued, A., Boyer, F., Douzi, B., and Cascales, E. (2015). The type VI secretion TssEFGK-VgrG phage-like baseplate is recruited to the TssJLM membrane complex via multiple contacts and serves as assembly platform for tail tube/sheath polymerization. PLoS Genet. *11*, e1005545.

Chang, Y.W., Rettberg, L.A., Ortega, D.R., and Jensen, G.J. (2017). *In vivo* structures of an intact type VI secretion system revealed by electron cryotomography. EMBO Rep. *18*, 1090–1099.

Chen, V.B., Arendall, W.B., 3rd, Headd, J.J., Keedy, D.A., Immormino, R.M., Kapral, G.J., Murray, L.W., Richardson, J.S., and Richardson, D.C. (2010). MolProbity: all-atom structure validation for macromolecular crystallography. Acta Crystallogr. D Biol. Crystallogr. *66*, 12–21.

Cianfanelli, F.R., Monlezun, L., and Coulthurst, S.J. (2016). Aim, load, fire: the type VI secretion system, a bacterial nanoweapon. Trends Microbiol. *24*, 51–62.

Clemens, D.L., Ge, P., Lee, B.Y., Horwitz, M.A., and Zhou, Z.H. (2015). Atomic structure of T6SS reveals interlaced array essential to function. Cell *160*, 940–951.

Durand, E., Nguyen, V.S., Zoued, A., Logger, L., Péhau-Arnaudet, G., Aschtgen, M.S., Spinelli, S., Desmyter, A., Bardiaux, B., Dujeancourt, A., et al. (2015). Biogenesis and structure of a type VI secretion membrane core complex. Nature *523*, 555–560.

Emsley, P., Lohkamp, B., Scott, W.G., and Cowtan, K. (2010). Features and development of Coot. Acta Crystallogr. D Biol. Crystallogr. *66*, 486–501.

Fernandez, I.S., Bai, X.C., Murshudov, G., Scheres, S.H., and Ramakrishnan, V. (2014). Initiation of translation by cricket paralysis virus IRES requires its translocation in the ribosome. Cell *157*, 823–831.

Fokine, A., Zhang, Z., Kanamaru, S., Bowman, V.D., Aksyuk, A.A., Arisaka, F., Rao, V.B., and Rossmann, M.G. (2013). The molecular architecture of the bacteriophage T4 neck. J. Mol. Biol. *425*, 1731–1744.

Forster, A., Planamente, S., Manoli, E., Lossi, N.S., Freemont, P.S., and Filloux, A. (2014). Coevolution of the ATPase ClpV, the sheath proteins TssB and TssC, and the accessory protein TagJ/HsiE1 distinguishes type VI secretion classes. J. Biol. Chem. *289*, 33032–33043.

Ge, P., Scholl, D., Leiman, P.G., Yu, X., Miller, J.F., and Zhou, Z.H. (2015). Atomic structures of a bactericidal contractile nanotube in its pre- and post-contraction states. Nat. Struct. Mol. Biol. *22*, 377–382.

He, S., and Scheres, S.H.W. (2017). Helical reconstruction in RELION. J. Struct. Biol. *198*, 163–176.

Ho, B.T., Dong, T.G., and Mekalanos, J.J. (2014). A view to a kill: the bacterial type VI secretion system. Cell Host Microbe *15*, 9–21.

Jobichen, C., Chakraborty, S., Li, M., Zheng, J., Joseph, L., Mok, Y.K., Leung, K.Y., and Sivaraman, J. (2010). Structural basis for the secretion of EvpC: a key type VI secretion system protein from *Edwardsiella tarda*. PLoS One *5*, e12910.

Kapitein, N., Bonemann, G., Pietrosiuk, A., Seyffer, F., Hausser, I., Locker, J.K., and Mogk, A. (2013). ClpV recycles VipA/VipB tubules and prevents non-productive tubule formation to ensure efficient type VI protein secretion. Mol. Microbiol. *87*, 1013–1028.

Kostyuchenko, V.A., Chipman, P.R., Leiman, P.G., Arisaka, F., Mesyanzhinov, V.V., and Rossmann, M.G. (2005). The tail structure of bacteriophage T4 and its mechanism of contraction. Nat. Struct. Mol. Biol. *12*, 810–813.

Krissinel, E., and Henrick, K. (2007). Inference of macromolecular assemblies from crystalline state. J. Mol. Biol. *372*, 774–797.

Kube, S., and Wendler, P. (2015). Structural comparison of contractile nanomachines. AIMS Biophys. *2*, 88–115.

Kube, S., Kapitein, N., Zimniak, T., Herzog, F., Mogk, A., and Wendler, P. (2014). Structure of the VipA/B type VI secretion complex suggests a contraction-state-specific recycling mechanism. Cell Rep. *8*, 20–30.

Kucukelbir, A., Sigworth, F.J., and Tagare, H.D. (2014). Quantifying the local resolution of cryo-EM density maps. Nat. Methods *11*, 63–65.

Kudryashev, M., Wang, R.Y., Brackmann, M., Scherer, S., Maier, T., Baker, D., Dimaio, F., Stahlberg, H., Egelman, E.H., and Basler, M. (2015). Structure of the type VI secretion system contractile sheath. Cell *160*, 952–962.

Kumar, S., Stecher, G., and Tamura, K. (2016). MEGA7: molecular evolutionary genetics analysis version 7.0 for bigger datasets. Mol. Biol. Evol. *33*, 1870–1874.

Leaver-Fay, A., Tyka, M., Lewis, S.M., Lange, O.F., Thompson, J., Jacak, R., Kaufman, K., Renfrew, P.D., Smith, C.A., Sheffler, W., et al. (2011). ROSETTA3: an object-oriented software suite for the simulation and design of macromolecules. Methods Enzymol. *487*, 545–574.

Leiman, P.G., Basler, M., Ramagopal, U.A., Bonanno, J.B., Sauder, J.M., Pukatzki, S., Burley, S.K., Almo, S.C., and Mekalanos, J.J. (2009). Type VI secretion apparatus and phage tail-associated protein complexes share a common evolutionary origin. Proc. Natl. Acad. Sci. USA *106*, 4154–4159.

Leiman, P.G., Chipman, P.R., Kostyuchenko, V.A., Mesyanzhinov, V.V., and Rossmann, M.G. (2004). Three-dimensional rearrangement of proteins in the tail of bacteriophage T4 on infection of its host. Cell *118*, 419–429.

Li, X., Mooney, P., Zheng, S., Booth, C.R., Braunfeld, M.B., Gubbens, S., Agard, D.A., and Cheng, Y. (2013). Electron counting and beam-induced motion correction enable near-atomic-resolution single-particle cryo-EM. Nat. Methods *10*, 584–590.

Lossi, N.S., Manoli, E., Forster, A., Dajani, R., Pape, T., Freemont, P., and Filloux, A. (2013). The HsiB1C1 (TssB-TssC) complex of the *Pseudomonas aeruginosa* type VI secretion system forms a bacteriophage tail sheathlike structure. J. Biol. Chem. *288*, 7536–7548.

Lovell, S.C., Davis, I.W., Arendall, W.B., 3rd, De Bakker, P.I., Word, J.M., Prisant, M.G., Richardson, J.S., and Richardson, D.C. (2003). Structure validation by Calpha geometry: phi, psi and Cbeta deviation. Proteins *50*, 437–450.

MacDonald, J.T., Kelley, L.A., and Freemont, P.S. (2013). Validating a coarse-grained potential energy function through protein loop modelling. PLoS One *8*, e65770.

Murshudov, G.N., Vagin, A.A., and Dodson, E.J. (1997). Refinement of macromolecular structures by the maximum-likelihood method. Acta Crystallogr. D Biol. Crystallogr. *53*, 240–255.

Pettersen, E.F., Goddard, T.D., Huang, C.C., Couch, G.S., Greenblatt, D.M., Meng, E.C., and Ferrin, T.E. (2004). UCSF Chimera–a visualization system for exploratory research and analysis. J. Comput. Chem. *25*, 1605–1612.

Pietrosiuk, A., Lenherr, E.D., Falk, S., Bonemann, G., Kopp, J., Zentgraf, H., Sinning, I., and Mogk, A. (2011). Molecular basis for the unique role of the AAA+ chaperone ClpV in type VI protein secretion. J. Biol. Chem. *286*, 30010–30021.

Planamente, S., Salih, O., Manoli, E., Albesa-Jove, D., Freemont, P.S., and Filloux, A. (2016). TssA forms a gp6-like ring attached to the type VI secretion sheath. EMBO J. *35*, 1613–1627.

Russell, A.B., Wexler, A.G., Harding, B.N., Whitney, J.C., Bohn, A.J., Goo, Y.A., Tran, B.Q., Barry, N.A., Zheng, H., Peterson, S.B., et al. (2014). A type VI secretion-related pathway in Bacteroidetes mediates interbacterial antagonism. Cell Host Microbe *16*, 227–236.

Sana, T.G., Berni, B., and Bleves, S. (2016). The T6SSs of *Pseudomonas aeruginosa* strain PAO1 and their effectors: beyond bacterial-cell targeting. Front. Cell. Infect. Microbiol. *6*, 61.

Vettiger, A., Winter, J., Lin, L., and Basler, M. (2017). The type VI secretion system sheath assembles at the end distal from the membrane anchor. Nat. Commun. *8*, 16088.

Wang, S., Ma, J., Peng, J., and Xu, J. (2013). Protein structure alignment beyond spatial proximity. Sci. Rep. *3*, 1448.

Yap, M.L., and Rossmann, M.G. (2014). Structure and function of bacteriophage T4. Future Microbiol. *9*, 1319–1327.

Zhang, K. (2016). Gctf: real-time CTF determination and correction. J. Struct. Biol. *193*, 1–12.

Zhang, X.Y., Brunet, Y.R., Logger, L., Douzi, B., Cambillau, C., Journet, L., and Cascales, E. (2013). Dissection of the TssB-TssC interface during type VI secretion sheath complex formation. PLoS One *8*, e81074.

STAR★METHODS

KEY RESOURCES TABLE

REAGENT or RESOURCE	SOURCE	IDENTIFIER
Bacterial and Virus Strains		
Expression strain *E. coli* B834(DE3): F − ompT hsdSB (rB − mB −) gal dcm met (DE3)	(Lossi et al., 2013)	N/A
Chemicals, Peptides, and Recombinant Proteins		
Vector for protein expression pET28-B1C1	(Lossi et al., 2013)	N/A
Deposited Data		
Cryo-EM map of *P. aeruginosa* TssB1C1 sheath	This paper, deposited at EMDB	EMD-3600
Atomic model of *P. aeruginosa* TssB1C1 sheath	This paper, deposited at PDB	PDB 5N8N
Software and Algorithms		
MOTIONCORR	(Li et al., 2013)	http://cryoem.ucsf.edu/software/driftcorr.html
Gctf	(Zhang, 2016)	http://www.mrc-lmb.cam.ac.uk/kzhang/Gctf/
RELION	(He and Scheres, 2017)	https://github.com/3dem/relion
ResMap	(Kucukelbir et al., 2014)	http://resmap.sourceforge.net/
Coot	(Emsley et al., 2010)	https://www2.mrc-lmb.cam.ac.uk/personal/pemsley/coot/
REFMAC	(Murshudov et al., 1997)	http://www.ccp4.ac.uk/
PHENIX	(Adams et al., 2010)	https://www.phenix-online.org/
MolProbity	(Chen et al., 2010)	http://molprobity.biochem.duke.edu/
Rosetta	(Leaver-Fay et al., 2011)	https://www.rosettacommons.org/software
PyMOL	Schrödinger, LLC	https://pymol.org/
UCSF Chimera	(Pettersen et al., 2004)	https://www.cgl.ucsf.edu/chimera/
RaptorX	(Wang et al., 2013)	http://raptorx.uchicago.edu/
PISA	(Krissinel and Henrick, 2007)	http://www.ebi.ac.uk/pdbe/pisa/
MEGA	(Kumar et al., 2016)	http://www.megasoftware.net/
RAMPAGE	(Lovell et al., 2003)	http://mordred.bioc.cam.ac.uk/~rapper/rampage.php

CONTACT FOR REAGENT AND RESOURCE SHARING

Further information and requests for resources and reagents should be directed to and will be fulfilled by the Lead Contact, Paul Freemont (p.freemont@imperial.ac.uk).

METHOD DETAILS

Expression and Purification of TssB1C1 Sheath

P. aeruginosa TssB1 and TssC1 proteins were produced in *E. coli* cells and purified as described previously (Lossi et al., 2013) with minor modifications. Cells were lysed by sonication and the buffer used during size exclusion chromatography was as follows: 50 mM Tris-HCl pH 9, 250 mM NaCl, 1 mM EDTA, 5 mM DTT giving longer and unfragmented tubes. For expression of TssB1C1 (with an N-terminally His_6 tagged TssB1) in *P. aeruginosa*, the corresponding sequences were cloned into the broad range vector pBBR1-MCS4 and the sheaths were purified using the conditions described above.

Negative-Stain Electron Microscopy

Purified TssB1C1 sheaths (0.1 mg/ml) were applied to a glow-discharged copper mesh EM grid overlaid with a continuous carbon support film. Samples were then negatively stained with 2 % (w/v) uranyl acetate and imaged using an FEI Tecnai 120 kV electron microscope operated at a magnification of 67,000×. Images were recorded at various defoci on a 2,048 × 2,048 pixel TemCam-F216 camera (TVIPS) using a dosage of ~ 30 e-/Å^2. The final pixel size of the resulting images is 2 Å/pixel.

Cryo-Electron Microscopy and Image Processing

Initial data collected at Diamond Light Source was critical in optimizing sample preparation. Full data collection on optimized TssB1C1 sheaths was performed at the Electron Microscopy facility of the MRC Laboratory of Molecular Biology, Cambridge. TssB1C1 sheaths (1 mg/ml) purified from *E. coli* cells were visualized on vitrified cryo-EM grids using an FEI Titan Krios electron microscope operated at an acceleration voltage of 300 kV and a nominal magnification of 59,000×. Sets of images were recorded with a sampling of 1.34 Å/pix on the FEI Falcon II direct electron detector operated in dose fractionation or "movie" mode with a cumulative dosage of 30 $e^-/Å^2$. Images were recorded at various defoci ranging from 1.0-2.5 μm using FEI EPU software for automated data-collection. A total of 1,405 "movie" mode image stacks were obtained. These image stacks were corrected for the effects of beam-induced motion using MOTIONCORR (Li et al., 2013). Motion-corrected sum images were produced using all recorded frames. Image Contrast Transfer Function (CTF) was determined by Gctf (Zhang, 2016), which enabled the selection of 1,280 out of 1,405 images whose Thon rings extend beyond or up to 4.5 Å resolution.

The helical single-particle analysis approach employed by RELION (He and Scheres, 2017) was used for image processing. Good 2D class averages of sheath segments were used as references for full auto-picking of the remaining 1,280 images that led to the identification of 107,157 helical segments. These segments were windowed into 350 x 350 pixels, have an overlap of ~ 93 % and therefore have an inter-box distance of ~ 7 % (33.6 Å) that corresponds to 10 asymmetric units. Once extracted, the helical segments were subjected to two rounds of 2D classification, giving 71,264 good segments for reconstructing the final 3D electron density map. Subsequent diffraction pattern analysis of several class averages gave preliminary values for the helical parameters of repeat distance, rise and twist that correspond to a 13-fold symmetric assembly. These values were inputted into RELION (program *relion_helix_toolbox*, option –*simulate_helix*) to generate a 13-fold pseudo-subunit model that has spheres placed in the defined helical lattice, for use as a preliminary 3D reference once low-pass-filtered to 30 Å.

A single round of helical 3D auto-refinement using motion-corrected sum images was performed to give an initial converged structure. During iterative refinement, no masking was carried out, C1 point-group symmetry was used and helical symmetry was locally searched and refined within a range of 0.2 Å and 3° for helical rise and twist, respectively. For out-of-plane tilts and in-plane rotations (psi) of extracted segments, we adopted search ranges of +/- 20° and +/- 15°, respectively, around their angular priors estimated from auto-picking. The reconstruction from 3D auto-refine was masked and post-processed, B-factor sharpening the reconstruction and correcting for the effects of convolution due to masking using phase randomization. Subsequent movie refinement and particle polishing gave "shiny" particles with improved signal-to-noise ratios. These were then subjected to another round of helical 3D auto-refinement, masking and post-processing to give the final correctly converged structure at 3.3 Å resolution based on the gold-standard 0.143 Fourier Shell Correlation (FSC) criterion. The mask used for post-processing only covers the central 30 % length of the box to exclude the top and bottom parts of the reconstruction that might be affected by slight alignment inaccuracies (He and Scheres, 2017). The final, refined helical parameters for rise and twist used to obtain the structure were 3.36 Å and -55.4°, respectively. However, based on the connectivity between neighboring TssB1C1 heterodimeric subunits, this helical symmetry is equivalent to a rise and twist of 20.2 Å and 27.7°, respectively. ResMap (Kucukelbir et al., 2014) was used to estimate the local resolution of the final structure.

Atomic Building and Modeling

The *V. cholerae* VipAB heterodimer (PDB 3J9G) was placed into the electron density map of the TssB1C1 sheath. Non-conserved residues were changed and a number of loops were rebuilt manually using Coot (Emsley et al., 2010). Iterative rounds of manual model building and automated refinement was performed in both reciprocal- and real-space using REFMAC (Murshudov et al., 1997) and PHENIX (Adams et al., 2010), respectively. This ultimately led to the successful tracing of ~ 89 % of the structure (132 residues from a total of 172 for TssB1 and 461 residues out of 498 in total for TssC1), which has good protein geometry and fit to the experimental density with small differences between FSC_{WORK} and FSC_{TEST} cross-validation curves (Fernandez et al., 2014), demonstrating that an optimal weight between the geometric restraints and density fit was used to prevent over fitting during refinement. The final refined atomic structure has a B-factor distribution that is in close agreement with the local resolution of the final 3D electron density map. The quality of the final atomic structure was assessed using MolProbity (Chen et al., 2010).

Models of TssB1C1 assemblies with 12-fold symmetry were generated in extended and contracted conformations based on the structures of *P. aeruginosa* R2 pyocin (PDB 3J9Q) and *V. cholerae* VipAB sheath (PDB 3J9G), respectively. Homology modeling of the N-terminal "arm" of TssB1 (residues 15-38) was based on the structural template provided by the R2 pyocin N-terminal "arm" (residues 2-25) that undergoes a conformational change in the extended state (PDB 3J9Q). Following loop modeling (MacDonald et al., 2013) in the region between the N-terminal "arm" and the folded portion of TssB1, an initial extended model of the TssB1C1 assembly was generated by re-aligning the modified TssB1C1 heterodimer to the constituent sheath subunits of R2 pyocin in the extended conformation. TssC1 residues 38-64 were removed from the sheath extended state due to clashes. The initial contracted and extended TssB1C1 sheath models with helical symmetry were then energy minimized using the Rosetta molecular modeling suite (Leaver-Fay et al., 2011).

Protein structures and maps were visualized using Coot (Emsley et al., 2010), PyMOL (Schrödinger, LLC) and UCSF Chimera (Pettersen et al., 2004). Figures were produced using PyMOL and UCSF Chimera. The movie showing sheath contraction was generated in UCSF Chimera using the "morph conformations" command.

Structural alignments were performed using RaptorX (Wang et al., 2013). Buried surface areas and interaction surfaces between protomers in the sheath structures and models were calculated using PISA (Krissinel and Henrick, 2007). Structures were

superposed in Coot (Emsley et al., 2010) and the corresponding root mean square deviation (RMSD) values were calculated using the SSM superpose function.

Bioinformatic Analysis

Protein sequences have been retrieved from the *Pseudomonas genome* database (http://www.pseudomonas.com) and NCBI (https://www.ncbi.nlm.nih.gov/). Sequence alignment of TssB and TssC proteins with their homologues in *V. cholerae* and *F. novicida* have been performed using MAFFT and alignment images were generated using the web server ESPript 3 (http://espript.ibcp.fr). For phylogenetic analysis, protein sequences were retrieved by a BLASTP search using concatenated sequences of TssB1C1, TssB2C2 and TssB3C3 from *P. aeruginosa* as query against the Kyoto Encyclopedia of Genes and Genomes database (KEGG) (http://www.genome.jp/tools/blast/). The sequence of *F. novicida* IglAB was also included. TssBC sequences were aligned using MUSCLE (MUltiple Sequence Comparison by Log-Expectation) and likelihood-based phylogenetic analysis was conducted using MEGA version 7 (Kumar et al., 2016).

DATA AND SOFTWARE AVAILABILITY

The 13-fold symmetric cryo-EM density map of the contracted sheath has been deposited in the Electron Microscopy Data Bank under accession number EMD-3600. The 13-fold symmetric atomic model built into the EM map has been deposited in the Protein Data Bank under accession code PDB 5N8N. The 12-fold symmetric extended and contracted models are available upon request.

Structure, Volume *26*

Supplemental Information

Atomic Structure of Type VI Contractile Sheath from *Pseudomonas aeruginosa*

Osman Salih, Shaoda He, Sara Planamente, Lasse Stach, James T. MacDonald, Eleni Manoli, Sjors H.W. Scheres, Alain Filloux, and Paul S. Freemont

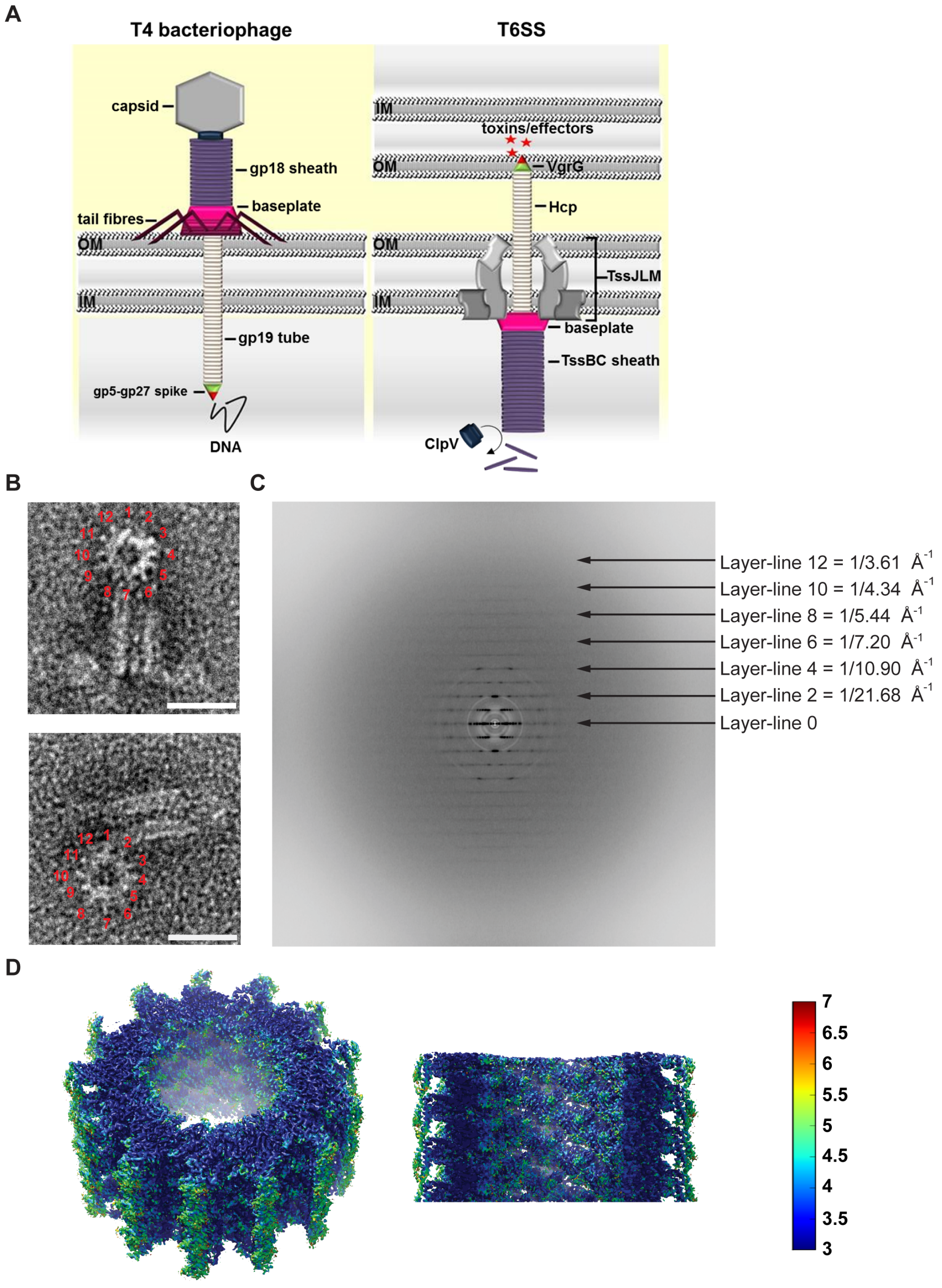

Figure S1. T6SS working model and assessing the quality of the electron microscopy data. Related to Figure 1. **A**. Working model of the T6SS assembly and its comparison with the T4 bacteriophage. The tail-like structure of the T6SS is highlighted: the Hcp tube in yellow, the VgrG spike in green and the TssBC sheath in purple. The AAA+ ATPase ClpV is shown in blue. Homologous structures found in the T4 phage are also shown and the same color code is applied. **B**. Top-views of TssB1C1 sheaths produced in *P. aeruginosa* showing 12-fold symmetry. The scale bars are 290 Å. **C**. Sum of Fourier transforms calculated from a subset of ~ 29,000 segmented sheaths in ~ 100 images showing layer lines in reciprocal space, extending up to 3.6 Å. **D**. Tilt-view and cut-away of a 162 Å long segment of the TssB1C1 sheath reconstruction. The structure is colored according to local resolution, ranging from 3 (blue) to 7 (red) Å.

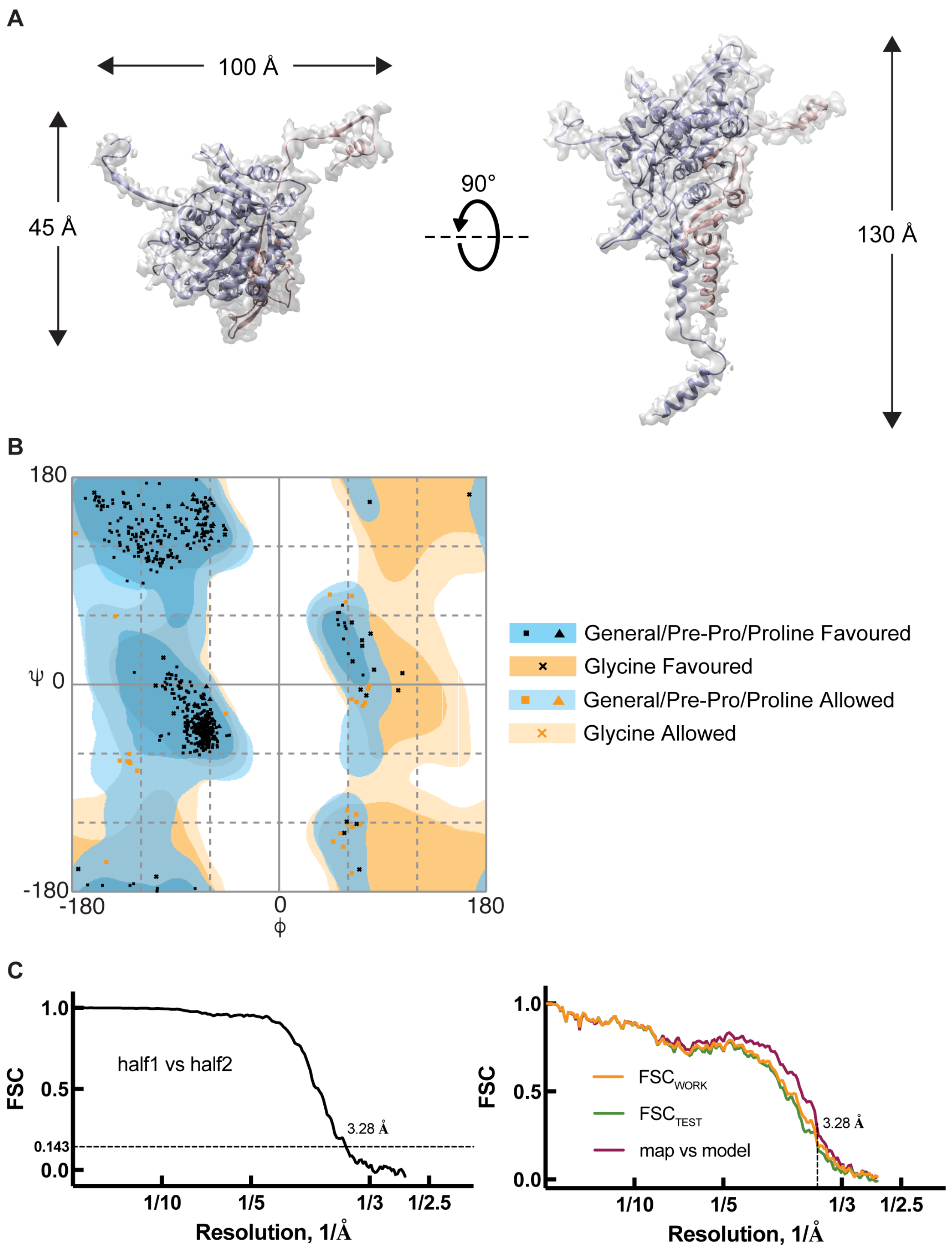

Figure S2. Atomic model of the TssB1C1 sheath. Related to Figures 1 and 2, and Table 1. **A**. Ribbon representation of the TssB1C1 heterodimer in its electron density map. Dimensions are indicated. **B**. Ramachandran plot of the TssB1C1 heterodimer. The Ramachandran plot was based on the atomic coordinates of the TssB1C1 heterodimer and was generated using the program RAMPAGE (Lovell et al., 2003). **C**. (Left) Fourier Shell Correlation (FSC) curve between the two halves of the final 3D electron density map. The resolution is 3.3 Å based on the gold-standard 0.143 FSC criterion. (Right) Cross-validation FSC_{WORK} and FSC_{TEST} curves demonstrate that an optimal weight during refinement was used to avoid over fitting. The FSC curve between the final 3D electron density map and the final refined atomic structure is also provided.

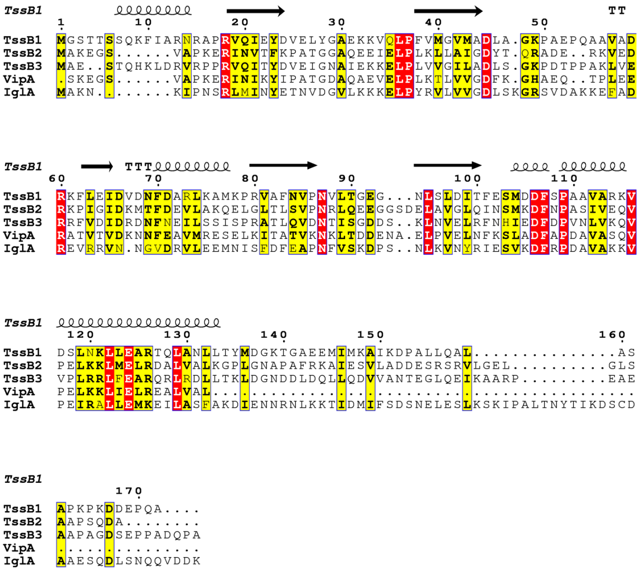

Figure S3. Sequence alignment of TssB proteins. Related to Figure 3. TssB protein sequences from *P. aeruginosa* were extracted from the Pseudomonas Genome Database; VipA and IglA sequences were retrieved from NCBI (https://www.ncbi.nlm.nih.gov/). Sequence alignments have been performed using MAFFT and the corresponding image generated using the web server ESPript 3 (http://espript.ibcp.fr). Secondary structure elements based on the *P. aeruginosa* TssB1 structure are displayed above the alignment.

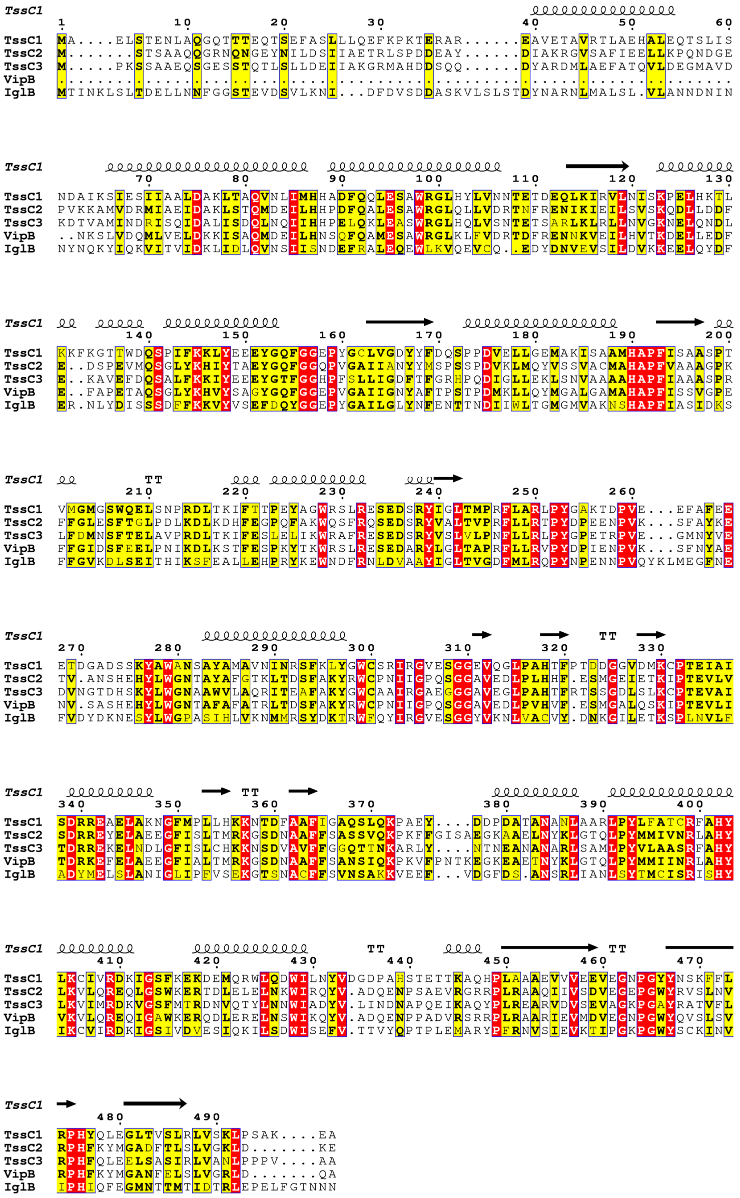

Figure S4. Sequence alignment of TssC proteins. Related to Figure 3. TssC protein sequences from *P. aeruginosa* were extracted from the Pseudomonas Genome Database; VipB and IglB sequences were retrieved from NCBI (https://www.ncbi.nlm.nih.gov/). Sequence alignments have been performed as described in Figure S3. Secondary structure elements based on the *P. aeruginosa* TssC1 structure are displayed above the alignment.

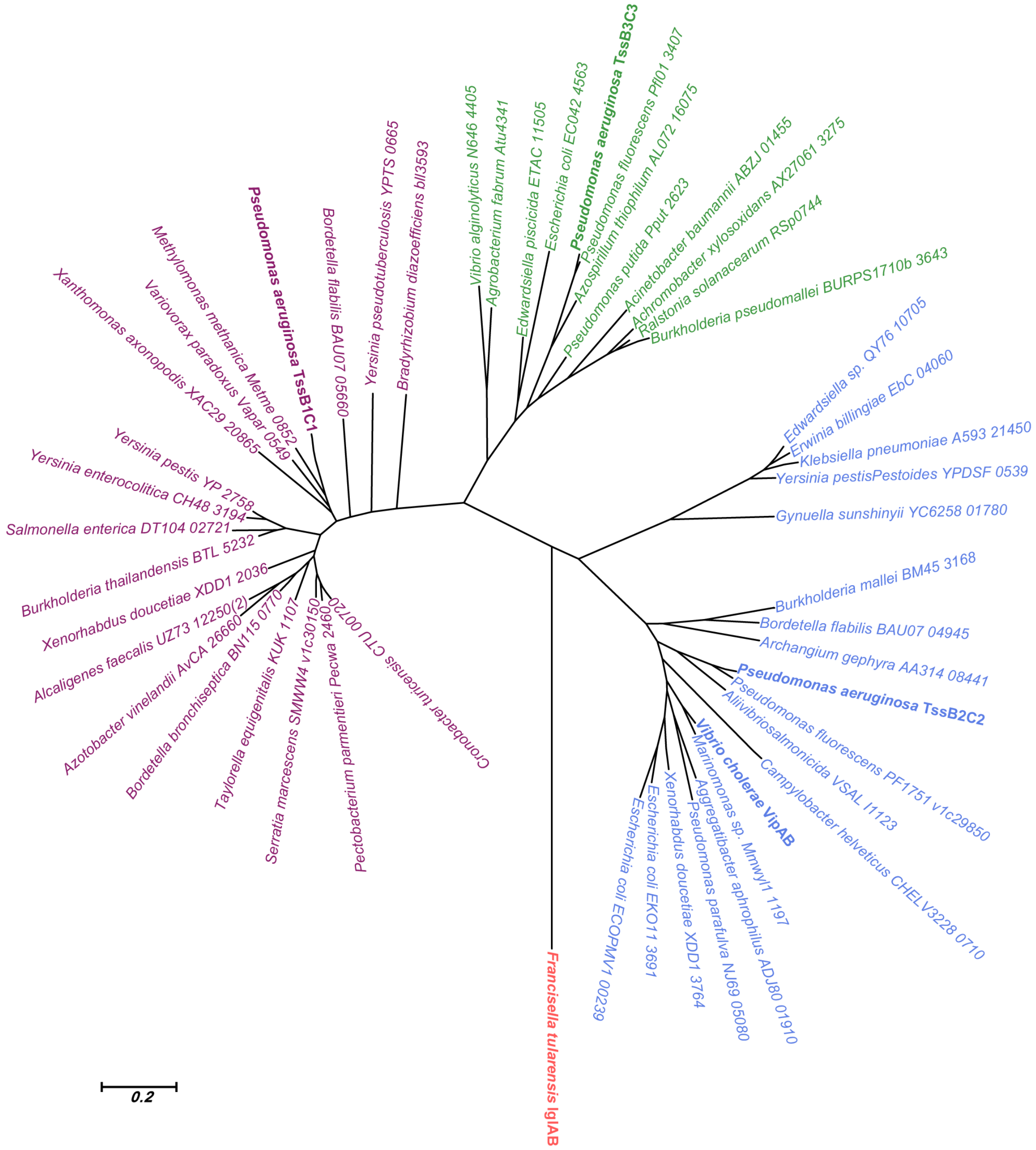

Figure S5. Phylogenetic analysis of T6SS sheath proteins. Related to Figures 3 and 4. The maximum-likelihood phylogenetic tree has been generated from 77 concatenated sequences of TssB-TssC. Concatenated sequences of the sheath proteins from *P. aeruginosa* (TssB1C1, TssB2C2 and TssB3C3), *V. cholerae* (VipAB) and *F. tulerensis* (IglAB) are highlighted in bold. The tree is drawn to scale, with branch lengths measured in the number of substitutions per site. All positions containing gaps and missing data were eliminated. Evolutionary analyses were conducted in MEGA7 (Kumar et al., 2016).

Table S1. Comparison of different sheath structures including the TssB1C1 models. Related to Figure 4.

	Sheath Interactions			Intra-strand Interactions			Inter-strand Interactions		
	Total Interface Area (Å^2)	Total No. H-Bonds	Total No. Salt Bridges	Total Interface Area (Å^2)	Total No. H-Bonds	Total No. Salt Bridges	Total Interface Area (Å^2)	Total No. H-Bonds	Total No. Salt Bridges
TssB1/TssC1 (13-mer)	9877.2	63	42	2671	21	15	3876.4	24	19
VipA/VipB (12-mer)	8756.7	87	40	2670.2	32	18	2593.5	20	8
IglA/IglB (12-mer)	9012.1	39	25	2895	15	8	2816.1	12	5
Contracted (12-mer)	9460.7	71	34	2565.5	21	10	3667.4	23	13
Extended (12-mer)	9732.1	69	32	3635.5	30	11	2637.5	13	8
